# Pattern of cesarean deliveries among women in an urban and rural district in Egypt

**DOI:** 10.4314/ahs.v22i4.43

**Published:** 2022-12

**Authors:** Monira M Gad, Amina A Mohamed, Heba M Abd El-Galil, Marwa M Mahgoub, Shaymaa M Ghazy, Mohammed SE Elsafty

**Affiliations:** 1 Department of Obstetrics and Gynecology, Faculty of Medicine (Girls), Al-Azhar University, Cairo, Egypt; 2 Department of Community Medicine, Faculty of Medicine (Girls), Al-Azhar University, Cairo, Egypt; 3 Department of Obstetrics and Gynecology, Faculty of Medicine, Alexandria University, Alexandria, Egypt; 4 Department of Obstetrics and Gynecology, Faculty of Medicine, Ain Shams University, Cairo, Egypt

**Keywords:** cesarean sections, CS rate, urban area, rural area, Egypt, obstetricians

## Abstract

**Aim:**

to compare patterns of delivery at an urban and a rural district in Egypt over 3 years.

**Methods:**

This retrospective study included 500 women and 50 obstetricians from each district from January, 2013 till December, 2015. Women answered a questionnaire about their deliveries. Obstetricians answered a questionnaire about their practice of CS.

**Results:**

CS rate in the rural district was 57.2% compared to 54.8% in the urban district in 2013. In 2014 and 2015, CS rates increased to 65.3% and 69%, respectively in the rural district compared to 56% and 57.7%, respectively in the urban district. 66% of obstetricians in the rural district performed CS for more than 50% of their patients compared to 76% of obstetricians in the urban district. 52% and 4% of obstetricians in the rural and urban districts, respectively, performed CS upon maternal request. 70.3% of women in the rural district who delivered by CS preferred to deliver vaginally. 51.4% of urban women who delivered by CS preferred to deliver vaginally. Level of education was the only factor showing statistical significance.

**Conclusion:**

CS rates increased over time with higher rates in the rural area. Level of women's education was the only factor affecting delivery choice.

## Background

In Egypt, rates of cesarean sections (CS) have risen to 52% as stated by Egypt Demographic and Health Survey (EDHS) in 2014 1. Regarding CS rates, Egypt now comes third after Dominican Republic and Brazil, having rates of 56.4% and 55.6%, respectively^2^. This global rise of CS rates is multi-factorial. Causes and factors that led to this rise include medical and non-medical indications like socio-economic and cultural causes along with a changes of risk factors in women over time^3^,^4^,^5^,^6^. Factors related to obstetricians and institutions at which women receive obstetric care has risen as independent risks for the increased CS rates^7^, ^8^,^9^.

The World Health Organization (WHO) stated that rates of CS above 10% were not associated with lower neonatal and maternal mortality^10^, ^11^. On the other hand, increased cesarean sections have an impact on health systems, is associated with higher maternal morbidity and could have side effects affecting further pregnancies^12^,^13^. According to the WHO, CS rates of 10 to 15% are optimal, yet countries do not have to seek this definite rate^14^. EDHS 2014 revealed that 90% of women received antenatal care and 87% of women delivered in health care facilities^1^, ^15^. 88% of deliveries were attended by obstetricians and 3% were attended by midwives, indicating the limited role of midwives in Egypt 1. In 2016, there were 2,600,173 deliveries in Egypt, of which a great proportion were conducted in health facilities^16^ and alongside, CS rates have alarmingly risen^17^. CS with non-medical reasons have also increased and were associated with increased maternal and neonatal morbidities^18^,^19^. Audits and routine monitoring of medical records by institutions are mandatory to review indications of CS hence keeping CS rates at an optimal level and avoiding CS done for non-medical indications^20^.

This study aimed to compare patterns of delivery at Al Montaza district, Alexandria as urban area and Kom Hamada district, which is a large rural area at El-Behira governorate in three consecutive years, to asses factors affecting selection of cesarean section by women and to identify obstetricians' views and practice of CS.

## Patients And Methods

This was a cross-sectional retrospective comparative study conducted at Kom Hamada District, a prominent rural area of El-behira Governorate, Egypt and Al-Montaza District, a prominent urban area of Alexandria Governorate, Egypt. Five hundred women who visited public local health offices in each district from January, 2013 till December, 2015 were randomly recruited. In addition, fifty obstetricians serving pregnant women in each district were recruited. Women who delivered either vaginally or by cesarean section or both between January, 2013 and December, 2015 were included in the study. Women who did not live in the studied districts or were non-Egyptians were excluded from the study. The study protocol was approved by the local ethics and research committee of Al-Azhar University, Faculty of Medicine (Girls' Section), Cairo, Egypt. All participants signed a written informed consent, ensuring confidentiality and privacy of participants.

Women were asked to answer a specially predesigned interview questionnaire to collect the following data: sociodemographic data like age, residence, educational level, occupation; past medical and obstetric history, mode of last delivery; indications of last cesarean section whether primary or repeat cesarean section; fetal and maternal outcome and any related complications. Obstetricians were also asked to answer a self-administered semi-structured questionnaire to identify their view for indications and complications of cesarean deliveries and their recommendations to reduce cesarean section rate. The caesarean rate was calculated as the number of caesarean births in each year divided by total number of deliveries in that year.

### Sample Size

All deliveries at the local health offices at each district were calculated. Sample size was calculated using Epi-Info version 7 with a 5% margin of error and a confidence level of 95%, with prevalence rate of CS of 52% was used. The yellow highlighted part will be removed.

Sample size was calculated from all deliveries at Al Montaza district (urban) which included eight health offices and Kom Hamada district (rural) which included 35 health offices using Epi-Info version 7 with a 5% margin of error and a confidence level of 95%, with prevalence rate of CS of 52%. Accordingly, the study included 500 women from each district which was slightly more than the minimum sample size required to compensate for women with incomplete data.

### Statistical Design

Continuous data were described in terms of mean ±SD, whereas categorical variables were described in number and percentage. Chi-squared test was used for the comparison of categorical variables while Student's t-test was used to compare between quantitative data. Significance level was taken at P-value ≤0.05. All analyses were performed using SPSS version 16 (SPSS Inc., Chicago, IL, USA).

## Results

In 2013, CS rate in Kom Hamada District (rural area) was 57.2% compared to 54.8% in Almontaza District (urban area). In 2014 and 2015, CS rates increased to 65.3% and 69% respectively in Kom Hamada District compared to 56% and 57.7%, respectively, in Almontaza District. CS rates showed a steady increase in both studied rural and urban areas with a higher percentage always seen in the studied rural area.

Fifty obstetricians from each district answered our predesigned questionnaire. Their ages were 43.1±11.3 years in the rural district and 41.4±5.7 years in the urban district, which was statistically insignificant. 74% were males in the rural district while 88.0% were females in the urban district, which was statistically significant. Most of the obstetricians were specialists in the studied rural and urban districts (72% and 78%, respectively) and worked in both public and private hospitals (80% and 86%, respectively). The differences were not statistically significant. 78% of obstetricians interviewed in the rural district expressed their favor of vaginal delivery compared to 82% of obstetricians in the urban district. Yet, it was statistically insignificant. In spite of their views, 66% of obstetricians in the rural district performed cesarean sections for more than 50% of their patients compared to 76% of obstetricians in the urban district, which was statistically significant. They reported that the most common indication for CS was previous CS; 70% and 46.0% in the rural and urban districts, respectively, which was statistically significant. Notable to mention that 12% of obstetricians in the urban district performed CS upon maternal request compared to no obstetricians in the rural district. 52% of obstetricians in the rural district would agree to perform CS upon maternal request (CSMR), while 16% would agree to perform CSMR after counselling the patient for vaginal delivery. On the other hand, 74% of obstetricians in the urban district agreed to perform CSMR after counselling for vaginal delivery, while only 4% agreed without counselling the patient for vaginal delivery. The difference was statistically significant.

96% of obstetricians both in rural and urban districts believed that proper counselling for benefits of normal delivery could help decrease CS rates, while 94% believed that proper antenatal care to predict suitable method of delivery and availability of continuous fetal monitoring is important for successful vaginal birth. 80% believed that doctors need to receive proper training for conducting safe vaginal delivery under different circumstances and 18% believed that obstetricians need protection from medico-legal issues they face when complications happen (Table 4).

**Table 4:** Obstetricians' views regarding principles to decrease CS rates

Studied group	a Physicians no. 100	
Items	No.	%
Good counseling with patient about benefits of normal delivery	96	96.0
Good antenatal care to predict the suitable mode of delivery	94	94.0
Provision of facilities necessary to determine mode of delivery such as partogram, fetal monitoring	94	94.0
Raising the awareness of physicians about the indications of C.S	90	90.0
Well training of doctors on good management of normal labour in different situations	80	80.0
Protection of doctors during legal liability if there were complications of normal labour	18	18.0
Increase fees of normal labour as it takes more time from the doctor	6	6.0
Good monitoring of hospitals by the government (or Ministry of Health and Population) to detect the rates and indications of C.S.	4	4.0
Using Media to raise the awareness of people about importance of good nutrition for young females to tolerate vaginal delivery later on with emphasis on benefits of normal delivery	4	4.0

a The percentage exceeded 100% because obstetricians gave multiple responses.

1000 women were included (500 from each district) in the study. Women recruited from the rural district were 27.7 ± 5.3 years old, compared to 28.5 ± 4.8 years old in urban region. 65.2% of women from rural district completed basic school while 58.6% of women from urban district completed university education. 80% of women from rural district were housewives compared to 68.8% of women from urban district. 66% of women from the rural district had current CS of which 49.6% was a primary CS. 84.8% had current CS of which 45.5% were primary. 99.4% of them had CS after first CS (Table 5). As shown in Table 6 and 7, the most common indication for CS was previous CS: 58.8% among women in the rural district compared to 54.5% among women in the urban district.

**Table 5:** General characteristic of studied women.

Studied females Items	Rural no.=500	Urban no.=500	Significant test & p-value
**Age** -Range -Mean ±SD	17–43 27.7±5.3	18–44 28.5±4.8	t. test NS
**Age groups(years) (n, %)** 17–35 ≥35	465(93%) 35(7%)	440(88%) 60 (12%)	X^2^ test 0.007*
**Level of education** **-Illiterate & read & write** **-Preparatory** **-Secondary** **-University**	66(13.2%) 31(6.2%) 326(65.2%) 77(15.4%)	37(7.4%) 42(8.4%) 128(25.6%) 293(58.6%)	X^2^ test 0.001*
**Occupation** **-House wife** **-Employed**	400(80.0%) 100(20.0%)	344 (68.8%) 156(31.2%)	X^2^ test 0.001*
**Parity -Para 1** **-Multipara (G2-G4)** **-Grand multipara** **(G5+)**	136(27.2%) 303(60.6%) 61(12.2%)	194(38.8%) 268(53.6%) 38(7.6%)	X^2^ test 0.001*
**Mode of current delivery** **-Normal vaginal delivery** **-Cesarean delivery** Primary Repeated	170(34%) 330(66%) **134(49.6%)** **196(59.4%)**	76(15.2%) 424(84.8%) **193(45.5%)** **231(54.5%)**	X^2^ test 0.001*
**Fetal outcome** **Full-term** **Pre-term**	463(92.6%) 37(7.4%)	394(78.8%) 104(21.2%)	X^2^ test 0.001*

**Table 6:** Indications of current CS among studied women in the rural area.

Studied females Indications	Rural no.=330
Previous Cesarean Section	194(58.8%)
Elective 1ry Cesarean Section	70(21.2%)
Emergency Cesarean Section	24(7.4%)
Fear of females from labour pain, pelvic floor injury and sexual problems	15(4.5%)
Post date	13(3.9%)
Hypertension	9(2.8%)
IUGR	5(1.4%)

**Table 7:** Indications of current CS among studied women in the urban area

Studied group Items	Females No. 424	
Indication	No.	%
- Previous CS	**231**	**54.5**
-Cephalo pelvic disproportion	**33**	**7.8**
-Mother choice	**27**	**6.4**
-Oligo hydraminos	**22**	**5.2**
- Abnormal presentations	**14**	**3.3**
- Twins	**13**	**3.1**
-Fetal distress	**10**	**2.4**
-Post maturity	**10**	**2.4**
-Failed trial	**9**	**2.1**
-Doctor told mother c.s is the best mode of delivery	**8**	**1.9**
-Primary and secondary infertility	**7**	**1.7**
- eclampsia	**7**	**1.7**
- Premature rupture of membranes	**6**	**1.4**
- Antepartum hemorrhage	**3**	**0.7**
-Intrauterine growth retardation	**2**	**0.5**
-Other	**22**	**5.2**

96.5% of women who delivered vaginally in the rural area were content with their mode of delivery while 3.5% preferred to deliver by CS. 70.3% of women in the rural district who delivered by CS preferred to deliver vaginally while 29.7% were content with their mode of delivery. Compared to women from the urban district, 84.2% of those who delivered vaginally were content while 15.8% preferred to deliver by CS and 51.4% of those who delivered by CS preferred to deliver vaginally while 48.6% were content (Table 8).

**Table 8:** Preferred mode of delivery among women according to current delivery.

Studied females	Rural no.=500		Urban no.=500		Significant test & p-value
Mode of delivery preferred	Vaginal delivery 170	CS 330	Vaginal delivery 76	CS 424	
- Normal -CS	164(96.5%) 6(3.5%)	232(70.3%) 98(29.7%)	64(84.2%) 12(15.8%)	218(51.4%) 206(48.6%)	X^2^ test 0.001*

Table 9 shows possible factors affecting preference for CS among women from rural and urban districts. Difference in level of education was the only factor that showed statistically significant difference (P value 0.001).

**Table 9:** Factors affecting preference for CS among studied women

Studied females Items	Rural no.=104	Urban no.=218	Significant test & p-value
**Age groups(years) (n, %)** 17–35 ≥35	95(91.3%) 29 (8.7%)	148(67.9%) 70 (32.1%)	X^2^ test NS
**Level of education** **-Illiterate & read & write** **-Preparatory** **-Secondary** **-University**	10(9.6%) 8(7.7%) 56(53.8%) 30(28.9%)	13(6.0%) 19(8.7%) 59(27.1%) 127(58.2%)	X^2^ test 0.001*
**Occupation** **-House wife** **-Employed**	76(73.1%) 28(26.9%)	147(67.4%) 71(32.6%)	X^2^ test NS

## Discussion

Our study showed that cesarean section rates have increased from 2013 until 2015 from 57% to 69% in the studied rural district compared to the studied urban district rising from 55% to 58%. It is worth noting that CS rates were always higher in the rural than the urban district. One study recorded a CS rate of 70.4% in Alexandria during 2017^21^. Comparing Egypt to other parts of the world, CS rate in Egypt was 51.8%, the highest in Africa; 47.9% in Iran, 47.5% in Turkey, 38.1% in Italy, 32.8% in United States, and 33.4% in New Zealand^2^. Arab countries had CS rates between 17.8% to 55.5%^22^–^25^. Other African countries had rates less than 5% and even as low as 2.3%^2^,^26^,^27^,^28^. Comparing to other governorates in Egypt, in the 2014 DHS reported CS rate of 70.4% in Kafr El-Sheikh, 76.6% in Port Said, 76% in Damietta and, on the other hand, CS rate was 26.2% in Matrouh^1^. In our study, 78% of obstetricians in the rural district compared to 82% in the urban district viewed vaginal delivery as the best delivery method, yet, 66% and 76% of obstetricians in the rural and the urban districts, respectively, performed CS for more than 50% of their patients per year. 70% of obstetricians from the rural district performed recurrent CS compared to 46% of obstetricians from the urban district. It is worth mentioning that 12% of obstetricians from the urban district performed CSMR compared to no obstetricians from the rural district. It is reported that obstetricians' favor for CS can affect pregnant women's choice for CS. Obstetricians do that by exaggerating medical care during normal vaginal birth, specially when women have great concerns and worries about possible complications of vaginal birth, mostly related to claimed higher perinatal mortality and morbidity. This incline to CS could be also driven by the higher tariff obstetricians receive for CS compared to vaginal delivery^29^. Moreover, the convenience of elective CS rather than unexpected timing and duration of vaginal birth has also been a pivotal factor for obstetricians' favor of CS^30^, ^31^. Furthermore, absence of national guidelines for normal vaginal birth and shift from public government hospitals to profit-minded institutions of the private sector has led to increased CS rates. The decreased role of midwives has caused a shift of obstetric practice from more comfortable settings with less restrictions on duration of childbirth to settings with higher technology and limited time, leading to greater clinician and patient anxiety^32^. In our study, a great majority of obstetricians (96% to 80%) believe that proper counselling of patients and antenatal care, availability of facilities like continuous fetal monitoring and proper training of doctors for management of labor could decrease cesarean section rates. Regarding studied women from both districts, 7% of women from the rural district compared to 12% from the urban district were above 35 years. There is a greater risk of congenital fetal malformations, hypertension, diabetes and increased use of fertility treatments in women with higher age leading to increased incidence of maternal and fetal morbidities which leads to higher CS rates^33^. In our study, 65% of rural women completed basic school education and 59% of urban women completed university education. 66% of rural women and 85% of urban women had cesarean sections, of which 50% and 46%, respectively were primary CS. More rural women whether delivered vaginally or by CS preferred vaginal delivery compared to urban women. One factor that appeared to affect preference for CS by women was level of education. As level of education became higher, preference for CS increased. A review by Jadoon et al.^34^ along with other studies^35^, ^36^ stated that women's educational status strongly predicts cesarean delivery. It was found that, after adjustment of confounding factors like age and parity, highly educated women who delivered by CS had strong medical indications and were less likely to deliver by CS for non-medical indications^37^.

## Conclusion

CS rates increased over time with higher rates in the rural area compared to the urban area. Level of women's education was the only factor that affected their choice of delivery.

## Figures and Tables

**Table 1:** Prevalence of cesarean section rates over 3 years of study

Year	Area	Vaginal delivery		Cesarean Section		Total
		No.	%	No.	%	
2013	-Rural -Urban	5667 12298	42.8 45.2	7572 14904	57.2 54.8	13239 27202
2014	-Rural -Urban	4400 11323	34.7 44.0	8283 14409	65.3 56.0	12683 25732
2015	-Rural -Urban	2575 9230	31.0 42.3	5737 12615	69.0 57.7	8312 21845

**Table 2:** General characteristics of studied obstetricians

Physicians Items	Rural no.=50	Urban no.=50	Significant test & p-value
**Age** -Range -Mean ±SD	28–73 43.1+11.3	29–55 41.4+5.7	t. test NS
**Sex (n, %)** -Female -Male	13(26.0%) 37(74.0%)	44(88.0%) 6(12.0%)	X^2^ test 0.001*
**Place of employment** -Public hospital -Private hospital -Both	4(8.0%) 6(12.0%) 40(80.0%)	6(12.0%) 1(2.0%) 43(86.0%)	X^2^ test NS
**Scientific degree** -Resident -Specialist -Consultant	8(16.0%) 36(72.0%) 6(12.0%)	5(10.0%) 39(78.0%) 6(12.0%)	X^2^ test NS

**Table 3:** Obstetricians' views and practice of CS

Physicians Items	Rural no.=50		Urban no.=50		Significant test & p-value
	no.	%	no.	%	
**Physician view towards CS** -Normal delivery is the best for delivery -CS is the best for delivery	39 11	78.0 22.0	41 9	82.0 18.0	X^2^ test NS
**The commonest indication for CS** -Previous CS -Cephalopelvic disproportion -Mal presentation -Maternal request -Fetal distress -Postdate	35 11 3 0 1 0	70.0 22.0 6.0 0.0 2.0 0.0	23 13 4 6 0 4	46.0 26.0 8.0 12.0 0.0 8.0	X^2^ test 0.02*
**Response to CSMR** -Disagree -Agree -Agree after advice with vaginal delivery at first	16 26 8	32.0 52.0 16.0	11 2 37	22.0 4.0 74.0	X^2^ test 0.001*
**Percentage of CS in practice /year** -Less than 15% -15–50% -More than 50%	6 11 33	12.0 22.0 66.0	0 12 38	0.0 24.0 76.0	X^2^ test 0.04*
